# Associations between focus constructions and levels of exhaustivity: An experimental investigation of Chinese

**DOI:** 10.1371/journal.pone.0223502

**Published:** 2019-10-09

**Authors:** Yu-Yin Hsu

**Affiliations:** Department of Chinese and Bilingual Studies, Hong Kong Polytechnic University, Hong Kong; Potsdam University, GERMANY

## Abstract

How various types of focus differ with respect to exhaustivity has been a topic of enduring interest in language studies. However, most of the theoretical work explicating such associations has done so cross-linguistically, and little research has been done on how people process and respond to them during language comprehension. This study therefore investigates the associations between the concept of exhaustivity and three focus types in Chinese (*wh*, cleft, and *only* foci) using a trichotomous-response design in two experiments: a forced-choice judgment and a self-paced reading experiment, both with adult native speakers. Its results show that, whether engaged in conscious decision-making or an implicit comprehension process, the participants distinguished *only*-focus and cleft-focus from *wh*-focus clearly, and also that there are specific differences between *only*-focus and cleft-focus in conscious decision-making. This implies that, in terms of the relationship between exhaustivity and the focus types under investigation, cleft-focus and *only*-focus behave very similarly during language comprehension despite the existence of some fine distinctions between them. In other words, the potential linguistic levels that exhaustivity encodes in Chinese cleft-focus render it more similar to *only*-focus than to *wh*-focus. These results are broadly in line with the semantic account that distinguishes cleft from *only*-focus, i.e., that cleft encodes exhaustivity in not-at-issue presupposition and *only*-focus encodes exhaustivity in at-issue assertion, while both express semantically encoded exhaustivity, triggering robust language-processing patterns that differ from patterns of *wh*-focus in Chinese.

## 1 Introduction

Cross-linguistically, sentences can highlight emphasized units (i.e., focus) prosodically (without syntactic reordering), or explicitly encode different types of focus through specific syntactic constructions [1–4]. The concept of exhaustivity exhibited in various focus constructions has mostly been treated either as semantically encoded, and possibly related to truth conditions, or as a conversational implicature that can be derived pragmatically from Grice’s (1975) [5] conversational maxims [6–8]. Language comprehension involves rapid integration of multiple levels of linguistic information, such as word meanings, phrase structures, and inferences about what speakers are trying to express, derived from specific linguistic forms in their utterances. While the concept of focus has been widely discussed in linguistics research, however, no consensus has been reached on how exhaustivity is represented in various focus types or processed in different focus contexts.

Theorists have argued that, cross-linguistically, identificational focus (sometimes referred to as contrastive focus) should be distinguished from information focus, on the grounds that these two focus types exhibit different exhaustivity effects and are expressed by different syntactic structures [2, 9–13]. The same line of argument holds that identificational focus exhaustively identifies a set of relevant entities (e.g., *only*-focus), whereas information focus refers to new and non-presupposed information (e.g., *wh*-question and answer pairs), and/or is underspecified for exhaustivity [14, 15]. Here, our main interest is in the exclusive exhaustivity expressed by *only*-focus. Theories supporting the parallelism of *only*-focus and cleft-focus in having exhaustive readings are often based on a logical association, as between (1) and (2), below. That is, the examples in (1) cannot be the logical consequences of the sentences in (2)[4], because when a speaker utters a sentence like (1a) or (1b), s/he asserts that ‘John saw the movie’ is the maximal and true statement, as compared to other alternative statements such as ‘John and Bill saw the movie’ or ‘John, Bill, and Susan saw the movie’. Thus, these types of foci are incompatible with additional members in the focalized domain (e.g., (3)). The same type of exhaustive reading has also been reported for *only*- and cleft-foci in Chinese [16, 17].

1
  - Only John saw the movie.
  - It was John who saw the movie.
2
  - Only John and Bill saw the movie.
  - It was John and Bill who saw the movie.
3
  *Only John saw the movie, and Bill did, too.
  *It was John who saw the movie, and Bill did, too.

To account for the sources of the exhaustive interpretations associated with *only*- and cleft-foci, some researchers have argued that the former express exhaustivity through semantic composition, as they are related to truth conditions, and that the same semantic exhaustivity also applies to cleft-focus (e.g., [2, 4]; for a review, see [18]).

However, some advocates of the semantic approach have pointed out that cleft-focus and *only*-focus may involve different types of exhaustive conditions (e.g., [19, 20]). For example, given that the cancellation of entailment targets content that is at issue, i.e., content relevant to answering the current question under discussion (QUD in [21]; [22]), we can distinguish at-issue content from not-at-issue content based on their compatibility of direct negation ([23] and the literature cited therein). As shown by example (4) (adopted from [23]), direct negation with *no*, as in (4B), denies the at-issue content of (4A); if one wants to deny the presupposition of (4A) *John has a dog*, using the direct negation *no* is infelicitous, as indicated by the # sign in (4B’). However, using a *but* response is felicitous when negating the presupposition of (4A), as shown in (4B”).

4
  A: John fed his dog.
  B: No, he didn’t.
  B’: #No, John doesn’t have a dog.
  B”: Well, he did feed a dog, but it wasn’t his dog.

Therefore, given the relationship between direct negation and (not-)at-issue content, the difference between *only*- and cleft-focus can be illustrated by the examples in (5) (adopted from [24]), in which negation is applied to test the exhaustivity of both *only*- and cleft-foci, and different acceptability judgments obtained for each.

5
  - Bob knew she invited Fred, but he didn’t know she only invited Fred.
  *Bob knew she invited Fred, but he didn’t know it was Fred she invited.

The contrast shown in (5) suggests that whereas *only*-focus encodes exhaustivity as a part of the assertion of at-issue content, and thus can be directly negated, cleft-focus semantically encodes exhaustivity differently: i.e., as an aspect of a presupposition rather than as part of the at-issue content [24]. Such differences have not been addressed by linguistic theories about Chinese *only*- and cleft-foci.

Nonetheless, some researchers have argued that differences of the type shown in (5) can be explained if cleft-focus encodes exhaustivity at the level of pragmatics, i.e., as a conversational implicature (similar to *wh*-questions), whereas *only*-focus encodes it in semantics and thus induces stronger exhaustive effects (e.g., [7, 23, 25]).

These analyses raise three important questions: During language comprehension, do native speakers of a given language express cleft-focus exhaustivity as strongly as they express *wh*-answer or *only*-focus exhaustivity? On what linguistic level(s)—i.e., semantics and/or pragmatics—is exhaustivity encoded in cleft-focus? And does cleft-focus differ across languages with respect to exhaustivity, and if so, how?

To date, these issues have mostly been discussed in the context of focus constructions in Hungarian and German. For example, to test the claim that Hungarian preverbal-focus items are similar to cleft-foci in English, insofar as they exhibit semantically encoded exhaustivity like English *only*-foci [2, 4], Onea (2009)[26] and Onea and Beaver (2011) [27] conducted truth-value judgment tasks. First, their participants were shown a picture describing an event involving two people (A and B); next, they heard a Hungarian sentence describing A alone, through the preverbal-focus or *only*-focus; and then, they had to choose among three possible responses, i.e., (a) *Yes, and B did, too*; (b) *Yes, but B did too*; and (c) *No, B did too*. Their analysis, which deemed all (a) and (b) answers to be non-exhaustive weak denial, and all (c) ones to be exhaustive, concluded that *only*-foci are relevant to semantic exhaustivity, whereas preverbal-focus items are compatible with non-exhaustive responses and therefore probably encode exhaustivity in pragmatics (see also [28]). Taking a slightly different approach, the current study builds upon and extends the explanatory power of the trichotomous design of responses, by not combining the results of (a) and (b) response types in our analysis. In light of arguments that the *but*-type of confrontation (e.g., the *Yes, but* response in our case), unlike direct negations such as *no*, can be a contradiction of not-at-issue content [23, 29] (for a discussion, see also [22, 30]). This property of the *but*-type of confrontation has therefore been taken into account in the current study.

Based on the results of a binary truth-value judgment task, Gerőcs et al. (2014) [31] found no significant difference between unmarked focus (i.e., *wh*-questions) and preverbal-focus in Hungarian, implying that both types of foci are related to pragmatic inference. The same authors then used an offline picture-matching task to examine exhaustive and nonexhaustive interpretations in preverbal and cleft-focus in Hungarian, with *only*-focus and *wh*-questions as the limiting cases. This established that the proportion of exhaustive responses to *wh*-questions was very low—in contrast to the results of their own online study—whereas the patterning of preverbal-focus differed significantly from those of both *only* and cleft-focus, meaning that preverbal focus might not express exhaustivity semantically. Taken together, the results of these studies suggest that *wh*-questions and preverbal-focus in Hungarian are more compatible with non-exhaustive contexts than that language’s cleft and *only*-foci are.

Drenhaus et al. (2011) [32] used a questionnaire survey and an event-related potential experiment to study German cleft and *only*-foci, and reported that violations of exhaustivity by cleft-foci, unlike those by *only*-foci, did not constitute semantic violations. Based on a picture-verification task, DeVeaugh-Geiss et al. (2017) [33] argued that the exhaustivity of German cleft-foci arises from presupposition; and Washburn et al. (2013) [34] used a naturalness-rating task to establish that, in English, both cleft-focus and *in-situ* contrastive focus can be compatible with non-exhaustive contexts, suggesting that their exhaustivity is derived from pragmatic implicature.

Liu and Yang (2017) [35] used an audiovisual Likert-scaled judgment task to test whether cleft-focus in Chinese expresses the same type of inference as three other sentence types (i.e., *only*-focus, plain focus, and simple sentences), and reported that cleft-focus differs from *only*-focus and plain focus, concluding that this challenges the pragmatic account. Then, they compared cleft and definite pseudo-cleft and reported that, unlike *only*- and *wh*-foci, cleft and definite pseudo-cleft patterned similarly, supporting the association of cleft with definiteness.

While [35] is the only recent study that favors a non-pragmatic account of Chinese cleft-focus’ definiteness, the question of whether cleft encodes exhaustivity semantically or pragmatically remains unsettled. Prior experimental results tend to support the view that *only*-focus semantically encodes exhaustivity, while *wh*-questions encode it pragmatically; but findings regarding cleft, and cleft-related foci have been mixed cross-linguistically. Very little theoretical or processing research on this topic has been carried out on Chinese languages, despite their profound typological differences from Hungarian and Germanic languages. Moreover, no studies have compared native speakers’ offline and online processing of these various focus types. The current study therefore examines whether and how exhaustivity is represented and processed across multiple focus contexts during adult native speakers’ Chinese language comprehension.

## 2 The present study

Like work on focus in other languages, research on Chinese focus has largely been devoted to theoretical semantic-syntactic accounts of various types of focus realization. While the term ‘exhaustive’ has been used in multiple ways by scholars describing types of Chinese focus, they generally agree that *zhiyou*-focus (the Chinese counterpart of English *only*-focus) and cleft-focus assert exhaustivity in semantics [36, 37]. Some recent studies have noted differences in the strength of exhaustivity across focus types, and in the Chinese case, have adopted either a semantic-compatibility view [38] or a syntactic-licensing/competition view [39, 40] to account for focus expressions’ various degrees of exhaustivity, as well as for their distributions within sentences. However, there is not yet any consensus on the linguistic levels that different focus types use to encode exhaustivity, or that native speakers use to interpret it.

Accordingly, the current study broadens the scope of previous linguistic studies by investigating the degrees of exhaustivity of three Chinese focus-constructions—*wh*-questions, *zhiyou* ‘only’, and cleft *shi… de*—and how they are processed during adult native Chinese speakers’ language comprehension. The marker *de* in the *shi… de* cleft construction in Chinese is located at the end of a cleft sentence (and it is often argued to be a type of sentence final particle; for more information, see Hole (2011) [16] and the literature cited therein). Therefore, target items in this study were all focalized subjects in the matrix subject position, so that the position and the length of target regions were both controlled. This study utilized 1) a forced-choice experiment with 66 participants, enabling direct observation of the preferred levels of exhaustivity they associated with particular types of focus; and 2) a self-paced reading experiment with a demographically similar, but non-overlapping group of 48 participants, to gauge their reactions to various types of responses under different focus conditions, as well as their processing of those types of focus and levels of exhaustivity, using a reading-time measure sensitive to language-processing effort [41].

The entire study was approved by, and conducted in accordance with, the ethical guidelines of the research review board of the Hong Kong Polytechnic University prior to the beginning of data collection.

Proceeding from two assumptions, 1) that the degree of exhaustivity of an assertion describing an event can be measured by participants’ comments about that assertion [26], and 2) that *only*-focus semantically asserts exhaustivity, we used simple non-focused declarative sentences and sentences with *only*-focus as the anchors of how native Chinese speakers processed *wh*-questions and cleft-foci, as well as of how three types of focus were associated with the concept of exhaustivity. It is also reasonable to expect that semantically encoded exhaustivity will be robust and systematic (e.g., *only*-focus), in contrast to pragmatically encoded exhaustivity (e.g., *wh*-focus).

The two experiments conducted as part of this study had three broad, interlinked aims: first, to increase our understanding of how representations of information change dynamically over time; second, to establish whether cleft-focus encodes exhaustivity on the semantic or pragmatic level; and third, to clarify whether and how the exhaustivity patterns associated with the focus constructions under investigation vary during language comprehension.

The experimental items in this study were all grammatical sentences encoding different types of focus and associated with different follow-up responses. Given that the amount of time spent reading can be deemed processing effort during comprehension [42–44], if comprehenders are sensitive to and process the (not-)at-issue differences triggered by *yes, but* responses, it is expected that longer reading time (RT) will be observed in trials associated with *yes, but* across focus conditions. This is because, theoretically, *but*-type responses target presupposed and not-at-issue contents, which should require more computation than responding to at-issue content does. In addition, measurement of RT by-region is taken in our study as an indication of the congruency of response and focus type, and used to compare the strength of exhaustivity encoded in the three focus constructions under investigation.

Prior to using a time-course sensitive experimental technique, our forced-choice experiment had three more immediate objectives: 1) to confirm the level of exhaustivity expressed by Chinese *wh*-questions, as compared to non-focus baseline sentences; 2) to examine whether cultural issues around expressing confrontation among the Chinese participants could have affected our study’s response paradigm; and 3) to empirically test native Chinese speakers’ conscious use of and judgment about focus constructions, for purposes of comparison with the results of our second experiment. Sections 3 and 4 will describe each experiment in turn, and Section 5 will briefly present our conclusions. The experimental items, de-identified participant demographic information, and data are available at https://osf.io/xg8rc/.

## 3 Forced-choice experiment

### 3.1 Method

#### 3.1.1 Participants

The participants in our first experiment comprised 66 native speakers of Chinese (54 female, 12 male; mean age ± SD: 21.5 ± 3.8 years) studying at a university in Hong Kong. All participants were right-handed, and had no left-handed relatives nor any self-reported history of brain damage. They responded to the study on a voluntary basis and received course credit for completing it. Given the IRB approval for this study (HSEARS20160811002), the need for the minor participants’ parent’s or guardian’s consent had been waived, because no such participants were involved.

#### 3.1.2 Materials

In this experiment, which used a within-subjects design, each participant completed 16 critical three-sentence dialogue trials interspersed among 43 filler trials, as more fully explained below. Three types of responses were manipulated to probe different levels of perceived exhaustivity. These were a *Yes,…* response, indicating acceptance (which would be compatible with non-focus or pragmatic implicature); a *Yes, but…* response, indicating a partial exhaustive reading related to the rejection of a not-at-issue presupposition; and a *No,…* response, indicating a strong rejection of the assertion, and associated with semantic exhaustivity [26, 27]. In each critical trial, the participant was asked to read a short conversation containing a context statement establishing a scenario (as illustrated in English in (6) and (7), see also Chinese example items in Table 1), and a target sentence containing a subject expressing one of the following four information types: i.e., 1) a non-focused bare subject (e.g., John in (6B)); 2) a *zhiyou* ‘only’-focused subject (e.g., *Zhiyou John*); 3) a cleft-subject (e.g., *Shi John*, ‘It was John that ….’); or 4) a bare subject offered in answer to a *wh*-question (e.g., John in (7B)). Then, the participant was asked to complete a Chinese dialogue by carefully choosing one response from among three, as exemplified in (6C). Some example stimuli are shown in Table 1.

**Table 1:** Example trials from Experiment 1 (forced-choice task). *Note*: Dashes indicate word boundaries, and square brackets in the English translations indicate words not uttered in Chinese. The abbreviation PERF in the glosses refers to perfective aspect, RC.marker refers to the marker of relative clauses, and POSS refers to possessor.

Information-structure Conditions		Responses
Non-focus bare subject	(以下是三個人的對話:) ‘Below is a conversation among three speakers:’ A:最近-限量款-球鞋-好-火! Zuìjìn-xiànliàng.kuǎn-qiúxié-hǎo-huǒ recently-limited.edition-sneaker-very-popular ‘Recently limited-edition sneaker[s] got really popular!’	C:—,小美-也-買到-了! —, Xiǎoměi-yě-mǎidào-le —, Xiǎoměi-also-buy-PERF ‘…, Xiaomei also got [a pair]!’ i.對啊,‘Yes,’ ii.對啊,不過‘Yes, but’ iii. 不對,‘No,’
(李紅Lǐhóng)	B:李紅-昨天-買到-了! Lǐhóng-zuótiān-mǎidào-le! Lǐhóng-yesterday-buy-PERF ‘Lihong got [a pair] yesterday!’	
*Wh*-answer subject	(以下是三個人的對:) A:今年-年終-抽獎?誰-抽中-了? Jīnnián-niánzhōng-chōujiǎng,shuí-chōuzhòng-le? this.year-year.end-lottery, who-win-PERF ‘Who won [the prize] at the year-end lottery?’	C:—,老李-也-抽到-了! —, Lǎolǐ-yě-chōudào-le —, Lǎolǐ-also-win-PERF ‘… Lao Li also won [it]!’ i. 對啊,‘Yes,’ ii. 對啊,‘Yes, but’ iii. 不對,‘No,’
(老王Lǎowáng)	B:老王-抽中-了! Lǎowáng-chōuzhòng-le Lǎowáng-win-PERF ‘Laowang won [it]!	
Cleft-subject	(以下是三個人的對話:) A:小欣-買-的-蛋糕-誰-偷-吃-了? Xiǎoxīn-mǎi-de-dàngāo-shuí-tōu-chī-le Xiǎoxīn-buy-RC.marker-cake-who-secretly-eat-PERF ‘Who secretly ate the cake that Xiaoxin bought?’ ’	C:—, 花花-也-偷-吃-了! —, Huāhuā-yě-tōu-chī-le —, Huāhuā-also-secretly-eat-PERF ‘… Huahua also ate [it]!’ i. 對啊,‘Yes,’ ii. 對啊,不過‘Yes, but’ iii. 不對,‘No,’
(是瑪莉It was Mǎlì)	B:是-瑪莉-偷-吃-的 Shì-Mǎlì-tōu-chī-de be-Mǎlì-secretly-eat-celft.marker ‘It was Mali who ate [it]!’	
*Only*-subject	(以下是三個人的對話:) A:昨天-誰-收到-了-新娘-的-禮物? Zuótiān-shuí-shōudào-le-xīnniáng-de-lǐwù yesterday-who-receive-PERF-bride-POSS-gift ‘Who got the gift from the bride yesterday?’	C:—, 晶晶-也-收到-了! —, Jīngjīng-yě-shōudào-le —, Jīngjīng-also-receive-PERF ‘… Jingjing also got [it]!’ i. 對啊,‘Yes,’ ii. 對啊,不過‘Yes, but’ iii. 不對,‘No,’
(只有小英 Only Xiǎoying)	B:只有-小英-收到-了! Zhǐyǒu-Xiǎoyīng-shōudào-le only-Xiǎoyīng-receive-PERF ‘Only Xiaoying got [the gift]!’

6
  Format of stimuli (for trials of non-focus, *only*-focus, and cleft-focus): (Here is a conversation among three speakers)
  A: There was a competition last week!
  B: I heard that John / only John / it was John who won a medal.
  C:
    - Yes, Bill also won a medal.
    - Yes, but Bill also won a medal.
    - No, Bill also won a medal.
7
  Format of stimuli (for *wh*-answer noun phrases): (Here is a conversation among three speakers)
  A: There was a competition last week! Do you know who won?
  B: John won a medal.
  C: (Same format as in (6))

As well as the 16 experimental trials and 43 filler trials featuring irrelevant types of dialogue, the experiment contained two practice trials aimed at familiarizing the participants with the experiment’s format. The 59 non-practice items were pseudo-randomized and manually checked so that no two trials with the same focus type were immediately adjacent to each other.

#### 3.1.3 Procedure

The first experiment was administered *via* online questionnaires. Before it commenced, the potential respondents read a description of it and indicated their consent to participation, and then provided their demographic information. Next, they received instructions regarding the appropriate completion of the dialogues. Then, after they had completed both practice trials, a message appeared on the screen telling them that the experiment was about to start. Each experimental trial was presented separately on the screen, and having read through the dialogue and chosen a response, the participant would press “Next”, and the following trial would appear. There was no time limit, so each session could be completed at the participant’s individual reading pace; in practice, the whole survey took each participant about 30 to 40 minutes.

#### 3.1.4 Analysis

To examine the relative levels of exhaustiveness exhibited in our four chosen types of constructions, we observed these 66 native speakers’ selection of a response (*yes*; *yes, but*; or *no*) that updated the current conversation with confronting information. Unlike in previous studies that used a binary (yes/no) forced-decision technique, we analyzed the impact of the three types of responses using a cumulative link mixed model. This included random intercepts for SUBJECT and ITEMS to fit the selection of three different types of RESPONSE, with the predictor FocusType (non-focus bare-subject, *wh*-answer subject, cleft-subject, and *only*-subject), using the clmm() function in the ordinal package [45] in R version 3.3.3 [46]. The dependent variable was used as an indicator of the levels of the participants’ acceptance/rejection of the exhaustivity of the foci being presented to them. The results were obtained based on likelihood-ratio tests of the model comparisons, and post-hoc comparisons were conducted using the lsmeans package of R [47].

### 3.2 Results and discussion

As shown in S1 Fig, the non-focus bare-subject and *wh*-answer subject conditions received the two lowest proportions of *no* responses, whereas participants responded to the cleft-subject and *only*-subject conditions using proportionally more *no* responses, and many fewer *yes* ones. Among all conditions, the cleft-subject received by far the highest proportion of *yes, but* responses.

Statistical analyses confirmed these observations. Specifically, there were significant effects of FocusType on the selection of responses (*wh*: B = 0.73, SE = .27, *p* = .008; cleft: B = 1.95, SE = .28, *p* <.001; *only*: B = 5.58, SE = .35, *p* <.001). As verified by post-hoc testing, both the *wh*-subject and the bare-subject conditions differed significantly from the *only*-subject (*p* <.001) and cleft-subject conditions (*p* <.001). Significant differences were also found between *only*- and cleft-subject conditions (*p* <.001) and between the non-focus bare-subject and *wh*-answer subject conditions (*p* = .04).

**Fig 1:**
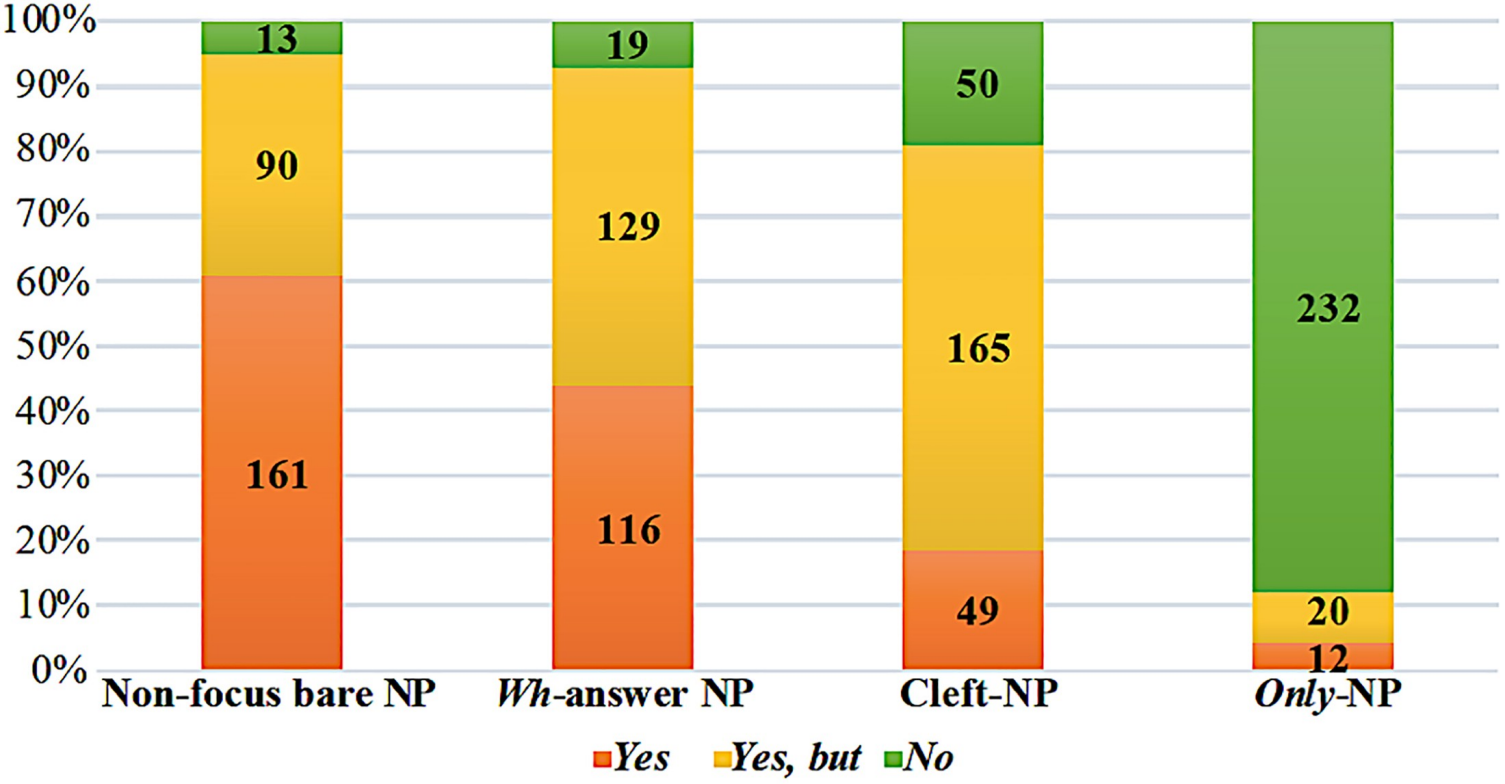
Responses to all four types of information structure. The numbers indicate the counts of each response type.

Taken together, the results of Experiment 1 illustrate two important points. First, that various strategies were used to provide confronting information by Chinese speakers, and that the choices among such responses were based on the specific information structures provided in the discourse. Because politeness is crucially important in Chinese culture, and thus likely to influence most conversations, it would be reasonable to expect that most of the responses across focus types would be either the positive *Yes*, or the positive, weak denial *Yes, but*. However, such a prediction was not borne out in the Experiment 1 results (see Fig 1).

Second, we found that different focus conditions tended to trigger different types of responses. *Wh*-answers and non-focus statements patterned more similarly, insofar as both had relatively high proportions of *yes* responses and much smaller proportions of *no* responses, despite being statistically different from each other. This is in accordance with the theoretical claim that the exhaustivity in *wh*-answers is encoded in conversational implicature, which may be under-recognized or undefined within discourse, and thus, we observed a much higher proportion of *yes* responses in association with *wh*-answers than in *only*- and cleft-foci. I am grateful to a reviewer of a previous draft of this paper for pointing out that the similar proportions of *yes* (43.9%) and *yes, but* (48.8%) responses in answers to *wh*-questions may be because answers to *wh*-questions allow either a “mention-some” or a “mention-all” reading, in the sense proposed by van Rooy (2004)[48]. It is to be expected that a mention-all reading will trigger more confrontations than a mention-some reading will. However, since the choice of one or the other of these readings depends on “whether a, and what kind of, human concern lies behind the fact that the question was asked” (page 406 in [48]), the choice of either *yes* or *yes, but* responses might equally be felicitous, depending on the speakers’ conversational priorities and the context where a conversation took place.

Crucially, in our study, the cleft-condition did not pattern similarly to either *wh*-answers or *only*-focus. The fact that the cleft-condition had the highest proportion of *yes, but* responses, and much smaller (yet similar) proportions of *yes* and *no* responses, suggests that the exhaustivity encoded in cleft may not be the pragmatic type; and instead—given that that *only*-focus encodes exhaustivity in assertion of at-issue content, and the different patterns between *only*- and cleft-focus that we observed—the cleft’s exhaustivity may be more relevant to assertion of not-at-issue presupposition.

Nonetheless, our results from Experiment 1 can still be compatible with either semantic or pragmatic accounts to assertive presupposition. That is, while the highest proportion of *yes, but* responses of cleft-focus in our study can be explained by the semantic account (as in [24]), some theories argue the word ‘but’ itself may bring pragmatic contrast to hint something that is different from the relevant content at-issue (e.g., [49]). Moreover, a forced-choice task does not allow participants to indicate felicitous alternatives. Also, it is well-known that metalinguistic judgments may not fully reflect language users’ real-time processing. To address these concerns, the following section uses results from a self-paced reading experiment to assess whether more robust distinctions can be observed among the three focus types when participants processed these focus constructions associated with each of the three types of responses during Chinese language comprehension.

## 4 Self-paced reading experiment

### 4.1 Methods

#### 4.1.1 Participants

In our second experiment, the participants were 48 native speakers of Chinese (36 female, 12 male; mean age ± SD: 23.7 ± 2.9 years); none of whom had taken part in the first experiment. Again, all were right-handed students at a university in Hong Kong who did not report having any left-handed relatives or any history of brain damage or speech or hearing impairment. All received an explanation of the study and its procedures, and gave their informed consent prior to its commencement. Each received HK$60 (about US$8) as an incentive to participate. Given the IRB approval (HSEARS20160811002), the need for the minor participants’ parent’s or guardian’s consent had been waived, because no such participants were involved.

#### 4.1.2 Materials

The second experiment was designed to capture how native speakers process conversations in which the basic information is expressed by one of three focus types, i.e., *wh*-focus, *only*-focus, or cleft-focus. The 18 critical trials (i.e., six different iterations of each focus type) were formatted for Linger software [50], which allows time-sensitive observation of readers’ sentence processing. In this case, six practice trials and 72 filler trials were also constructed, all following the same dialogue format as the critical ones. As shown in Table 2, each critical trial started with a leading context, establishing a scenario, and this was followed by a response that showed one of three degrees of acceptance/negation (e.g., C’s utterances in Table 2: *yes* (Y); *yes, but* (YB); and *no* (N)).

**Table 2:** Example trial from Experiment 2 (self-paced reading task). *Note*: Presentation units are separated by slash marks. Square brackets in the translation indicate words not uttered in Chinese.

The Leading Context	Follow-up responses
(以下是三個人的對話:)	C:/ **RESP_Region4_**/ SUBJ_Region5_ / **ALSO_Region6_** / **VP_Region7_**
‘In what follows, you will see	C_Y_: / 對啊, / 老李 / 也/ 抽到了 。
a conversation among three speakers:’	/ Duìa,/ Lǎolǐ/ yě/ chōudào.le
A:今年/年終/抽獎,/誰/抽中/了?	/ Yes,/ Lǎolǐ/ also/ get.PERF
Jīnnián/niánzhōng/chōujiǎng,/shuí/chōuzhòng/ le?	‘Yes, Lǎolǐ also got [one].’
this.year/year.end/lottery,/who/win/PERF	C_YB_:/ 對, 不過/ 也/ 抽到了/ 。
‘Who won the prize of the year-end lottery?’	/ Duì, bùguò/ Lǎolǐ/ yě/ chōudào.le
B:**老王_Region1_** / 抽中_Region2_ / 了_Region3_	/ Yes, but/ Lǎolǐ/ also/ get.PERF
Laowáng/ chōuzhòng/ le!	‘Yes, but Lǎolǐ also got [one].’
Laowáng/ get/ PERF!	C_N_:/ 不對,/ 老李 / 也/ 抽到了 。
‘Laowáng got [it]!’	/Bùduì,/ Lǎolǐ/ yě/ chōudào.le
	/No,/ Lǎolǐ/ also/ get.PERF
	‘No, Lǎolǐ also got [one].’

The regions under investigation are speakers B and C’s utterances in each experimental trial. The regions that we measured RT (i.e., Regions 1 to 7) are marked in Table 2. We focused on each native speaker’s overall RT for all seven of these regions collectively, as well as his/her RT within each of them. However, we were particularly interested in the effects observed in the following four regions (marked in bold in Table 2): Region 1, the focused subject, which expressed one of the three focus types; Region 4, i.e., one of the three possible response types in speaker C’s statement (RESP.); Region 6, which contained the word ä¹? *ye* ‘also’, confirming the presence of confronting information; and Region 7, because it immediately followed the other three regions, enabling us to observe specific information-processing effects and potential spillover effects [51, 52].

#### 4.1.3 Procedure

During the experimental session, each participant was seated in front of a computer. Because the experimental material incorporated a ‘click-to-proceed’ element that prevented the participants from reading ahead, and thus from understanding any whole trial until they had finished reading it, it was possible to isolate their reactions to specific regions within trials.

Trials were presented on-screen in the format shown in Table 2, i.e., with the target sentence (B) containing a subject expressing one of the three focus types, followed by one of the three possible responses ascribed to speaker C. The sentences in each trial were shown in non-cumulative, moving-window, self-paced reading paradigm [41], whereby all presentation units in a trial are replaced with dashes, and the participant presses a key to mask the current unit and reveal the next one. Upon completing six practice trials, each participant was asked to read the 96 critical and filler trials carefully at their natural reading pace. At the end of each trial, he/she was shown a comprehension question designed to elicit information about the scenario as a whole, rather than about the aspects of the trial that were specifically under investigation. The whole procedure took each participant between 25 and 45 minutes to complete, not including three required 5-minute breaks. The participants’ reaction times for each presentation unit were recorded to allow us to estimate the processing effort of reading comprehension [41].

#### 4.1.4 Analysis

Our analyses excluded the times that the participants took to read 1) the filler trials, and 2) the presentation units that occurred before speaker B’s statements in the critical trials. We also excluded outliers for each participant and item, i.e., reading times for the main trials’ critical units that differed by more than two standard deviations from that participant’s mean for that presentation region. Then, the remaining RTs were log-transformed to approximate a normal distribution for purposes of analysis.

The accuracy of the participants’ responses to the comprehension questions was analyzed through generalized linear mixed models, generated using the glmer() function of the lme4 package [53] in R version 3.3.3 [46], with the predictors being FocusType and RESPONSE. Reading time was analyzed using linear mixed models with random intercepts for PARTICIPANTS and ITEMS [54], and the models were fit with the following predictors: FocusType (wh; only; cleft), RESPONSE (yes; yes, but; no), and the presentation REGION. Model evaluation was *via* using log-likelihood tests, which examined whether the inclusion of additional predictors and/or their interaction contributed significantly to the statistical model fitting. Pairwise post-hoc Tukey’s comparisons were then conducted with R’s multcomp package [55].

### 4.2 Results

#### 4.2.1 Accuracy

The participants in the second experiment responded correctly to 95.4% of all items. In the three focus categories, this broke down as 96.1% correct in the *wh*-subject condition; 94.8% in the cleft-subject condition; and 94.1% in the *only*-subject condition. There were no significant differences in the accuracy of the participants’ responses to the comprehension questions across conditions, and no interaction effects (χ^2^s<7.37, *p*s >.1).

#### 4.2.2 Reading time

After the removal of outliers, 5,640 observations remained for analysis. Standard deviations for the random effects in the saturated model were Participants:.205; Items:.032; and Residual:.226. REGION, RESPONSE, FocusType and the interaction of RESPONSE and FocusType were used to examine processing effects on participants’ (log-transformed) reading time.

Using RESPONSE-*yes* and FocusType-*wh* as the baseline for studying 1) speakers’ reactions to unexpected confronting information and 2) the level of exhaustivity of focus, we found significant fixed effects of both REGIONS (*p* <.001; see below for by region analyses) and RESPONSE (*no*: B = -.01, SE = .01, *p* = .046; *yes, but*: B = .01, SE = .01, *p* = .292). Post-hoc testing verified the significance of the differences between *no* and *yes, but* responses (*p* = .006): i.e., *yes, but* triggered longer reading time than *no* responses did. However, post-hoc tests indicated that the differences between *yes* and *no* responses (*p* = .11) and between *yes* and *yes, but* responses (*p* = .54) were not significant. There was no interactive effect of FocusType and REGION on the overall results of reading time.

**Fig 2:**
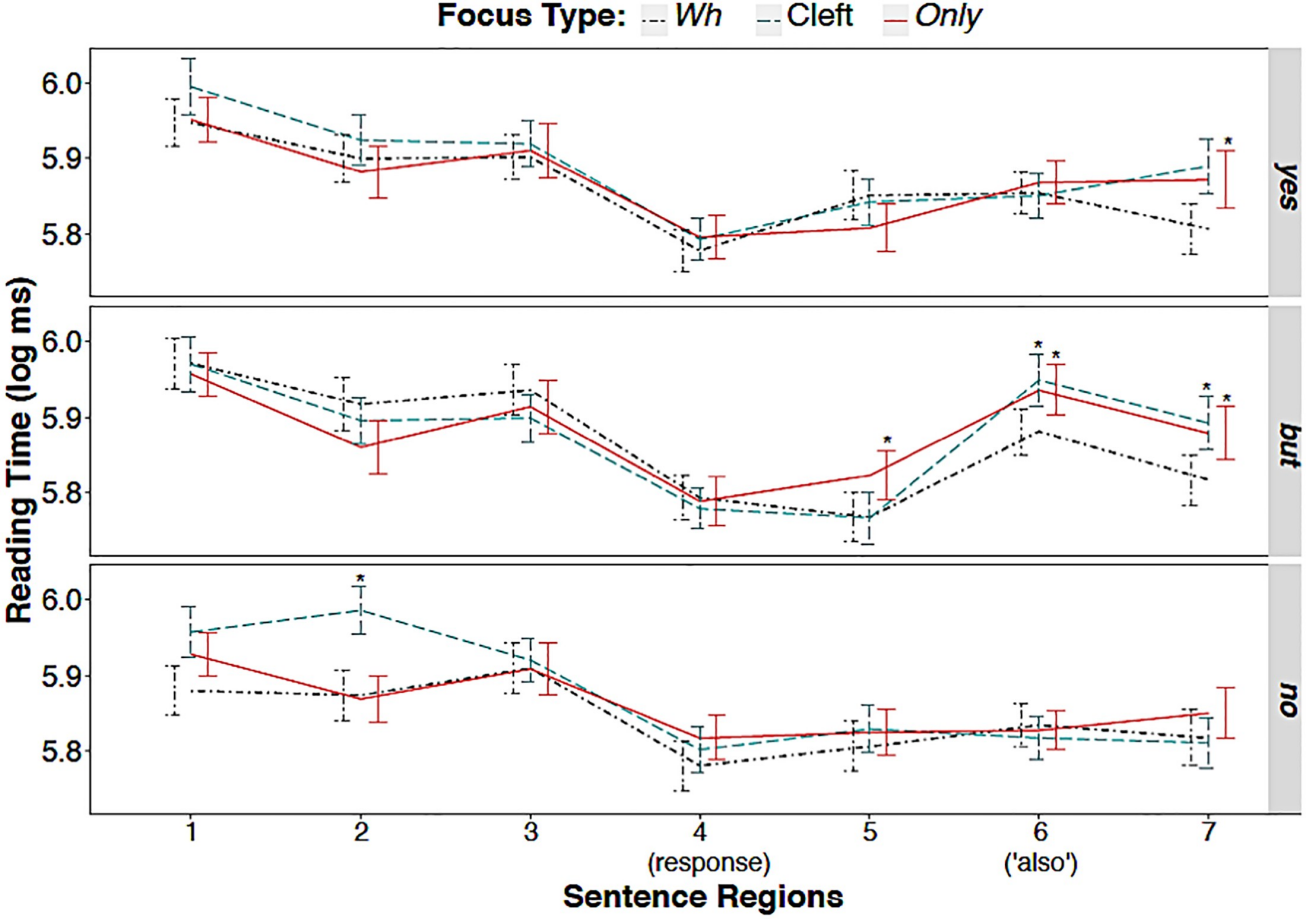
Region-by-region mean reading time of sentences by focus type and response type. *Note*: Regions showing a significant effect are indicated with asterisks. Error bars show ± .5 standard errors from the mean.

Next, we observe RT by region; the line plots in Fig 2 show region-by-region mean RTs for sentences in *wh*-, cleft-, and *only*-focus, categorized by response type (yes; yes, but; no). From this, we can observe that subjects (Region 1) in the cleft condition (the green long dash line) led to longer RTs than *wh*-answer subjects or *only*-subjects did, when the cleft was associated with *yes* and *no* responses (see Regions 1 and 2). No obvious differences in RTs across focus conditions were found at the end of the leading context (Region 3) or in the region of responses (Region 4), but differences became more prominent after the readers had processed the response, i.e., in Regions 5 through 7. Statistical results support this observation.

Using *wh*-answers and *yes* responses as the baseline, a mixed model of the participants’ RTs in Region 1 revealed no fixed effects of FocusType or RESPONSES, and no interaction effects. However, in Region 2, it indicated some interaction effects that caused cleft-condition to be read slower, particularly when it was associated with *no* responses (B = .11, SE = .04, *p* = .004). Similar patterns were also identified in the relationships between cleft-condition and the other two response types, but these were not significant (*yes*: B = .07, SE = .04, *p* = .095; *yes, but*: B = .03, SE = .04, *p* = .424). No significant fixed effects of FocusType or RESPONSE were found in Region 2.

In Regions 3 and 4, our analysis found no significant fixed or interactive effects of FocusType and RESPONSE on reading time. However, in Region 5, there were (marginally) significant fixed effects of RESPONSE on RTs, with both *yes, but* and *no* responses being associated with shorter reading times than the baseline (*yes, but*: B = -.08, SE = .03, *p* = .012; *no*: B = -.06, SE = .03, *p* = .076). Post-hoc testing identified differences between the responses *yes, but* and *yes* (*p* = .03). Though no fixed effects of FocusType were found in this region, some interaction effects were revealed: notably, combinations of *only*-focus with a *yes, but* response were processed more slowly than the baseline condition (B = .09, SE = .04, *p* = .042).

In Region 6, the interaction of RESPONSE and FocusType had significant effects. Specifically, while RTs for the focus types under investigation were all read faster when the response was *no*, significant differences were found between the interaction of cleft-foci and response *yes, but* (B = .11, SE = .03, *p* <.001), on the one hand, and on the other, the interaction of *only*-foci with the same type of response (B = .11, SE = .03, *p* <.001). Post-hoc testing further revealed that cleft-foci were processed slower in combination with *yes, but* responses than they were in combination with either *yes* ones (*p* = .012) or *no* ones (*p* <.001). *Only*-foci with *yes, but* responses were also processed slower than those with *no* responses (*p* <.001). No fixed effects of RESPONSE or FocusType were found in this region.

Lastly, in Region 7, FocusType had significant effects, but RESPONSE did not. Specifically, *only*-focus and cleft-focus were both associated with longer RTs than the baseline condition (*only*: B = .06, SE = .02, *p* = .010; cleft: B = .05, SE = .02, *p* = .057). Post-hoc testing revealed significant differences between *only*-focus and *wh*-answer (*p* = .026). The observed RT for *only*- and cleft-focus were all slower when the responses were *yes* and *yes, but* than when they were *no*, but post-hoc testing showed only marginal differences between *yes, but* and *no* responses (*p* = .075); and no significant interaction effects of RESPONSE and FocusType were found.

### 4.3 Discussion

As reported in section 4.2.2, there were fixed effects on RTs of both sentence regions and response types. Before we discuss the by-region results, in terms of the characteristics of three types of responses, it is worth noting that trials associated with *yes, but* responses indeed triggered significantly longer RTs than trials of *no* responses. In line with the theoretical assumption that the direct denial *no* is used to reject at-issue content, whereas *but*-type rejection targets not-at-issue presupposition, our results suggest that the native speakers we sampled were sensitive to the specific information associated with *yes, but* responses during language comprehension.

Across all four regions of special interest in Experiment 2’s critical trials, most fixed effects on reading time were found 1) immediately after the region of the focalized subject, and 2) in and immediately after the region of the word *ye* ‘also’. This is consistent with prior findings about spillover effects [51, 52]. Our finding that readers tended to process trials related to *no* responses faster suggests that such responses differ fundamentally from the other two response types we studied. In other words, as previously discussed, one possibility is that a direct denial *no* is associated clearly with exhaustive assertion, whereas the other two response types may either be essentially of the same type as each other during reading comprehension (e.g., weak denial [27]), or not be sensitively reflected by the RTs of the speakers we sampled during language comprehension—although these two types of responses were treated differently in the explicit judgment task in our first experiment. With respect to focus types, our results show that the sampled native speakers interpreted and processed cleft-focus and *wh*-answer focus rather differently; therefore, if *wh*-answers express exhaustivity in conversational implicature, it can be expected that cleft-foci encode exhaustivity differently.

Our by-region analysis also yielded some interesting findings. First, no meaningful differences were found between the *only*- and cleft-foci, and subjects in both these conditions required much more processing time than *wh*-foci did. Previous processing studies have likewise found that cleft-words in sentences take longer to read than non-cleft-words, and this has been taken to mean that cleft-foci require more comprehension effort, which in turn is probably related to the processing of exhaustive interpretations (e.g., [56–58]), and our results further indicated that it is related to the exhaustivity encoded in the not-at-issue presupposition. The similarity between cleft-subjects and *only*-subjects identified in our study may be a further indication that the relative difficulty of comprehending cleft- and *only*-subjects probably has the same source in both cases. It should also be noted that such processing efforts are not necessarily due to the phrasal complexity of the subject units. That is, in the region immediately after the focalized subjects, items across conditions were processed in a similar way until the reader processed the region ‘also’, which confirmed the piece of confronting information.

The fact that we found no differences in the effect of FocusType on reaction time between the response region and the immediately adjacent following region requires some explanation. In part, it might be due to the exhaustivity that cleft- and *only*-foci both exhibit; however, they differ insofar as the former express not-at-issue, presupposed exhaustivity and the latter assert at-issue exhaustivity, which in turn may prompt readers to accept a perceived agreement under both these conditions. Similarly, after reading the answer to a *wh*-question, readers may not expect a confronting statement. It is also possible that, in response regions and the regions immediately following them, because the participants had not yet obtained the information conveyed by the whole of speaker C’s statement, they tended to accept units faster if their meanings followed general cooperative conversational principles.

Interestingly, in the region where the word *ye* ‘also’ occurred, we observed differences in RTs across response types and focus types; that is, the participants’ reading of *only*- and cleft-focus materials was slower than their reading of the baseline *wh*-answer focus in the response conditions of *yes, but* and *yes* (see Fig 2). This suggests that native Chinese speakers processed *wh*-focus differently from the other two foci. Since *only*-focus semantically encodes exhaustivity, we expected that, after they read the additive adverb *ye* ‘also’, readers might experience more surprise and/or expend greater processing effort, resulting in longer RTs for the responses *yes* and *yes, but*. As S2 Fig. shows, *only*- and cleft-focus conditions under *no* responses were processed similarly to *wh*-answer focus conditions, whereas with responses of *yes, but*, and *yes*, cleft- and *only*-foci were processed slower than *wh*-focus conditions.

Taken together, these results indicate that the respective relations of *only*- and cleft-focus to exhaustivity were more similar to each other during language comprehension than we predicted. In light of the widely accepted theoretical claim that *wh*-focus encodes exhaustive readings in pragmatic implicature, our study suggests that exhaustivity is encoded in cleft not through pragmatics, but (like *only*-focus) in line with semantic encoding.

## 5 Conclusion

This study’s survey, based on Chinese data, of the relations among focus types and exhaustivity has tried to clarify some theoretical debates through the use of processing tasks. Specifically, its results regarding the association between exhaustivity and three focus types, obtained *via* a forced-choice judgment task and a self-paced reading-processing task, show that—whether engaged in conscious decision-making or an implicit process—native Chinese speakers do not always differentiate between *only*-focus and cleft-focus. Task-specific differences provide further interesting insights: with our offline study showing that *wh*-, cleft- and *only*-foci were significantly different from the baseline (i.e., non-focus sentences) and among themselves, but our online study indicating that the most important difference was between *wh*-focus and *only*/cleft-focus. Based on the results of both experiments, we may conclude that, in terms of their relationship to exhaustivity, cleft-foci in Chinese were processed much like *only*-foci; whereas *wh*-foci—perhaps due to contributions from pragmatics—pattern differently from *only*-foci in terms of the computation of exhaustive associations at a different linguistic level.

Moreover, while both cleft- and *only*-foci were processed similarly in our study’s implicit-comprehension task, different response preferences were revealed by the explicit (forced-choice) task. That is, while both cleft- and *only*-focus prompted significantly more *no* responses than the non-focus baseline and *wh*-focus conditions, the participants preferred to confront cleft-focus with *yes, but* responses, whereas with *only*-focus, they preferred to use *no* responses. Such a distinction, together with the findings from our self-paced reading experiment, support the semantic account of cleft’s exhaustivity—whereby the assertion is in the sphere of presupposition, and not at issue—in contrast to *only*-focus, which encodes exhaustivity as a part of the assertion, i.e., within the at-issue content [24].

This study also has some cross-linguistic implications for linguistic theory in terms of processing focus constructions. With the help of both explicit and implicit language-processing tasks and Chinese data, our results constitute an important contribution to ongoing debates about the nature of cleft-focus, and help clarify how Chinese speakers use and interpret three types of focus constructions during real-time language comprehension. While the semantic presupposition account of cleft-focus as in [24] also associates cleft with definiteness, the current study was not designed to examine this aspect. However, our results and those reported in [35] on cleft and definite pseudo-cleft complement each other in providing new insights into the general properties of cleft construction.

## Supporting information

S1 Fig Responses to all four types of information structure. (TIFF)

S2 Fig Region-by-region mean reading time of sentences by focus type and response type. (TIFF)
