# Supplementary figures and images for: Associations between focus constructions and levels of exhaustivity: An experimental investigation of Chinese
Source: PLoS One. 2019 Oct 9;14(10):e0223502. doi: 10.1371/journal.pone.0223502 (PMC6785076; doi:10.1371/journal.pone.0223502)

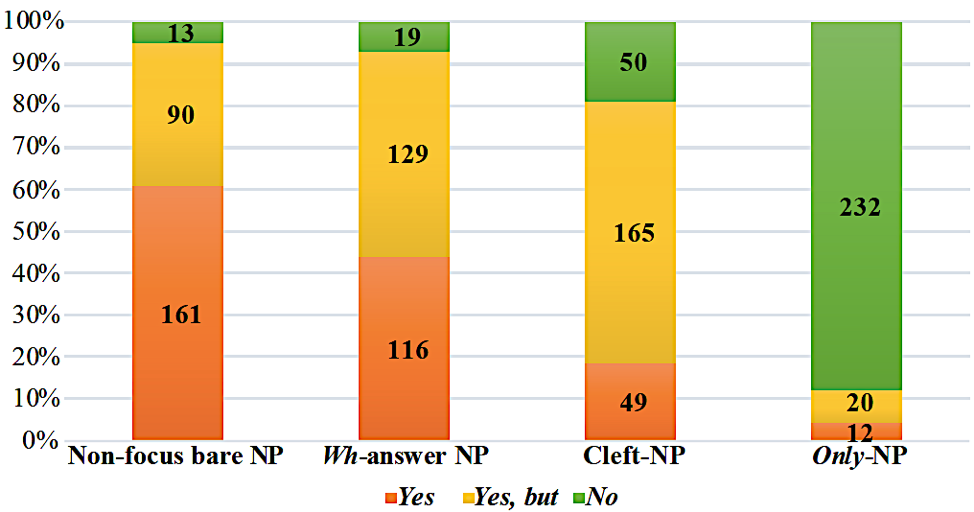

Supplement: S1 Fig — (TIFF) [file pone.0223502.s001.tiff]

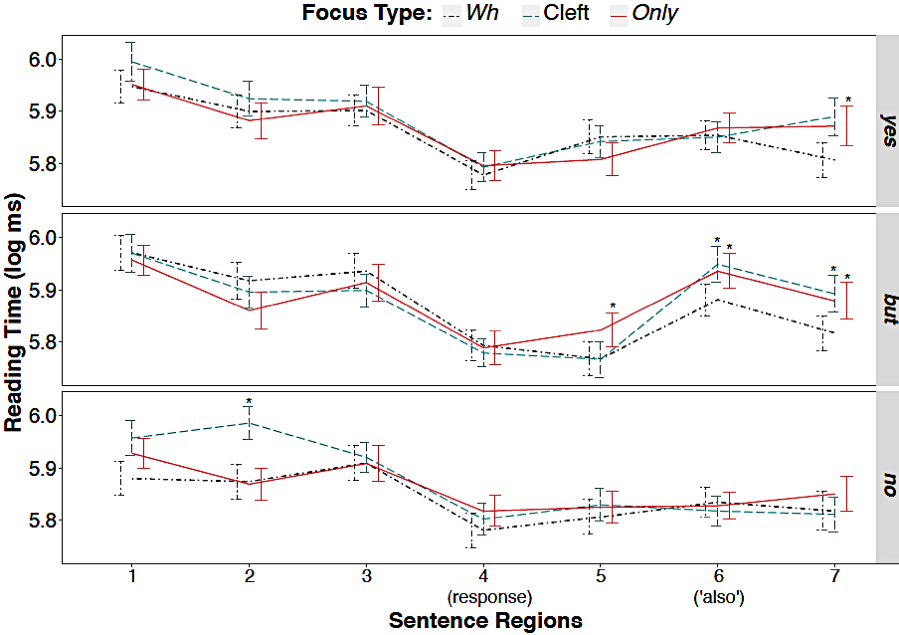

Supplement: S2 Fig — (TIFF) [file pone.0223502.s002.tiff]
